# Supplementary material for: Facial femininity and perceptions of eating disorders: A reverse-correlation study
Source: PLoS One. 2021 Aug 6;16(8):e0255766. doi: 10.1371/journal.pone.0255766 (PMC8345843; doi:10.1371/journal.pone.0255766)
Supplement: Attachment — Submitted filename: ReverseCorr_DouglasBalasGordon_Response_r2.pdf [file pone.0255766.s002.pdf]

Response to Reviews

We would like to thank all three of our reviewers for their thoughtful comments on the latest draft of our manuscript. We were pleased to see that they were very positive about this version of the text and we hope that these revisions have adequately addressed the issues they raised here. Below we have reproduced their narrative comments about the current draft, with our responses in boldface text.

Journal Requirements:

Please review your reference list to ensure that it is complete and correct. If you have cited papers that have been retracted, please include the rationale for doing so in the manuscript text, or remove these references and replace them with relevant current references. Any changes to the reference list should be mentioned in the rebuttal letter that accompanies your revised manuscript. If you need to cite a retracted article, indicate the article's retracted status in the References list and also include a citation and full reference for the retraction notice.

We have reviewed the reference list and incorporated the new citations suggested by our reviewers (noted below in response to their suggestions to do so). Otherwise, we have also corrected reference formatting in a few instances to be consistent with PLoS' instructions to authors.

Reviewer's Responses to Questions

We have omitted the Reviewers' responses to these questions in this document because there were no additional comments accompanying their answers. Below, we reproduce their comments to the authors in full, with point-by-point responses to each suggestion that indicate how we have edited the text to incorporate their feedback.

6. Review Comments to the Author

Reviewer #1: 1.Summary

Commentary on the revised version of the research report "Facial Femininity and Perception of Eating Disorders: A Reverse-Correlation Study". In the current version, the author did not substantially change the layout of the manuscript; therefore, the research report's description would be very much the same. Briefly, the authors used reverse correlation and FaceGen stimuli database to study whether facial femininity affects the probability that the face would be classified as belonging to a person who suffers from Eating Disorders (ED+ when yes, ED- when not). Authors also studied whether such ascribed characteristic is affected by the race (White or Black) of the face (stimuli). They found that when noise is applied, only faces of the Black race perceived as ED+ are also perceived as more feminine (by an independent group of on-line raters). Therefore, the authors demonstrated that race affects the way raters utilize facial cues when assessing eating disorders. In the second study, however, when they used faces

manipulated in facial femininity, both the Black and White race stimuli that were feminized, were more likely classified ad ED+.

The authors highlight the importance of their study as a "cornerstone" of a promising research field. The field would study whether facial appearance may affect the probability ("likelihood") of a person being perceived (and eventually mistreated) as likely or unlikely "victim" of an eating disorder, based solely on their appearance. They show that being of certain origin may affect the probability of being perceived as suffering from a certain illness (ED, in this very case). Authors also took the first step in identifying the traits that serve in such "folk classification" (sex-typicality).

2. Review of revisions

The authors took all my suggestions and objections into consideration and either extended/shortened the manuscript to address them, or replied why I might have been wrong. I appreciate their patience. Therefore, I recommend the paper for acceptance because, after the revisions, I can more clearly see its potential impact. The authors also state their willingness to proceed on this topic.

They added or extended the following sections within the manuscript:

They re-wrote the abstract to emphasize that they studied "perceptual biases regarding facial appearance" and if they affect the diagnosis based on the race of the stimuli face. They also extended the introduction to stress that they studied (racially-specific) bias itself and did not identify the alleged "kernel of truth" in such a classification.

Throughout the description of methods, the authors also extended the section on the FaceGen database. I appreciate that. The extended description substantially improved my image on the utility of the database.

Thank you - we're glad to hear that this provided useful context.

The authors also edited the section on the image analysis. While they kept the section on the effect of image background/foreground colouration, they shortened the section on the effect of root mean square contrast.

In experiment 2, the authors added another short description of the stimuli processing within FaceGen. Authors also added the ethics statement and changed the term "Eating disorder" to ED (and of course, they explained the abbreviation).

Authors solved the technical issue of data-inaccessibility (regardless of whether it was a failure of mine or the OSF itself). They also cited the statistic software they use so that I could check the data and analyses. However, in my opinion, they only provide the JASP files for the Image

statistical analysis. For the rest of the analyses, I could find only the raw MTurk data and classification images.

We apologize - these files should be available in our OSF repository at present.

Coming to the Response to Reviews section, authors replied in detail to my notions and suggestions, bringing further justification to their changes (or their decisions not to change the text according to the requests).

I accept their reply on my primary objections: I would like to thank them for explaining the study's actual goal and demonstrating that I was wrong in my suggestions. They explain what it is not a good idea to link the paper to evolutionary psychology. The revised version is much clearer regarding studying perceptual stereotypes based on facial traits and race (see also above).

I also accept that it would be extraordinarily complicated and ethically questionable to use photos of actual patients who suffer from eating disorders. In light of the manuscript's update, my suggestions on the use of different stimuli and study design are no more relevant. Also, the authors are right that reverse correlation (RC) is not a rare technique. My notion regarded just its usage within the field of face perception. Now, it is much clearer that both the goals and the whole "paradigm" of the current research report are much further from evolutionary psychology that I've been thinking. RC, therefore, seems a useful approach within the study of perceptual biases.

We appreciate the reviewer's thoughtful commentary about these issues and are glad to hear that our revisions helped clarify the key goals of our study.

They also claim that my suggestion to identify which features of the classification image affect the classification is partly resolved by the Image statistical analyses. Otherwise, they argue that capturing the aspects of texture that led to stimuli classification is above the article's scope of the article and exceeds the capacity and abilities of image analysis.

3. Suggestions:

Therefore, I have only several minor objections, which could, in my opinion, be solved easily:

Thank you for these additional comments - below we describe how we have edited the text to address the remaining suggestions.

Page 2: "...their femininity may affect observers' beliefs about their eating behaviour" I would add: "only under specific circumstances (manipulation of stimuli)". -> The abstract has been modified upon my request. My request, however, did not address the difference in results between the first and second experiment—my apologies.

We have revised this as suggested by the reviewer.

Page 5/References: Authors should add the reference to the following citation: Birhane & Guest, 2020 (currently missing in References).

Thank you. We have included this in the revised references.

Page 8: "That is, given this particular sample of faces described in terms of their 3D shape and 2D surface properties, what is a compact dimensional model of the data that captures the correlational structure of the data? The resulting model makes it possible to assign spatial coordinates to each face such that groups of faces can be characterized in terms of their distribution within this space." The second sentence, in my opinion, do not answer the first question. Consider writing something like: "That is, given this particular sample of faces described in terms of their 3D shape and 2D surface properties, the compact dimensional model of the data based on the faces makes it possible to assign spatial coordinates to each face such that faces can be characterized in terms of their distribution within this space." Of course, if I miss the important message of the sentences, keep the original version.

Thank you. We have adopted this suggestion in the revised text.

Page 12: "...covaried with the appearance characteristics associated with disordered eating by our first group or observers." Did you mean "first group of observers"?

We did. Thank you! This has been corrected in the revised text.

Page 12/References: Authors should add the reference to the following citation: JASP, 2018 (currently missing in References).

We have added this to the references.

Page 20/References: Authors should add the reference to the following citations: Fink et al., 2006; Matts et al., 2007; Mitteroecker et al., 2015 (currently missing in References).

We have added these to the references as well.

Throughout the text, authors should unify the citation of the American Psychological Association data (either 2015 or 2013).

We have corrected these inconsistencies.

Reviewer #2: The authors did an excellent job in addressing reviewer comments. I only have a few minor additional comments.

Abstract

Please briefly define what the reverse correlation technique is in the abstract

We have included a short description of the technique in the revised abstract.

Instead of saying “above-change levels” please include the relevant statistics

Our understanding is that specific statistical results are usually reserved for the main text of the paper, while the abstract is meant to serve as a more concise description of key outcomes. As such, we have edited the abstract to more clearly communicate this result, but only report the statistical tests we used and their outcomes in the relevant results section.

Introduction

It is good that you explained the reverse correlation technique, but I would do it before you introduce the term. Perhaps refrain from introducing until it is explained.

We have edited the text to minimize references to the technique before we explain its use.

I found the explanation of the reverse correlation technique to be confusing

We apologize for the lack of clarity. We have attempted to re-work this part of the manuscript to make it more clear how reverse correlation paradigms work and why it is useful in this case.

Please elaborate why peer-identification of EDs is important and biases may be problematic – e.g., that peers could refer individuals to treatment,

We have attempted to expand upon this point in the revised draft.

Methods:

I would minimize the use of acronyms – e.g., say MTurk instead of AMT workers, reverse correlation instead of RC

We have adopted this suggestion throughout the text.

Discussion:

Please discuss in more detail the prevalence in men vs. women with EDs (i.e., what percentage of EDs is in men vs. women? Does differ by the ED?)

This does vary by eating disorder - we have briefly included some of these statistics in the revised text.

Reviewer #3: Thank you for the opportunity to review this manuscript. This is an interesting topic that can be considered by readers. The Introduction section was very well prepared and comprehensively provides what is already known on this subject. The results are very interesting. However, after reviewing the manuscript, I have some minor comments:

1. I would recommend expanding the Discussion section as to how some of the results of the study could be implemented rather than a repeat of what was already discussed.

Thank you for this comment, we have attempted to briefly offer some speculations along these lines in the discussion.

2. References are missing in several places in the discussion.

We have included these in the revised text.

3. How these results compare to previous results is not discussed.

We have attempted to clarify how these results relate to previous findings, though to some extent this is a relatively new avenue of inquiry regarding how facial appearance and the perception of eating disorder diagnoses may be correlated.
