# Supplementary material for: Facial femininity and perceptions of eating disorders: A reverse-correlation study
Source: PLoS One. 2021 Aug 6;16(8):e0255766. doi: 10.1371/journal.pone.0255766 (PMC8345843; doi:10.1371/journal.pone.0255766)
Supplement: Attachment — Submitted filename: ReverseCorr_DouglasBalasGordon_ResponsePLOS.docx [file pone.0255766.s001.docx]

**Response to Reviews**

**We would like to thank our reviewer for their thoughtful comments regarding our manuscript. Below, we have reproduced their comments in full and responded point-by-point to their suggestions and indicated how we have revised the text in response. Throughout, our responses are in boldface and the original review is in unbolded text.**

Reviewer #1: 1. Summary

The research report “Facial Femininity and Perception of Eating Disorders: A Reverse-Correlation Study” applied the approach of the Reverse Correlation (which I find novel and rare) to examine, whether some concrete facial cues are associated with the perception of Eating Disorders (ED). The research focused on the problem of raising ED prevalence in contemporary US College population. It used artificial stimuli from FaceGen database and two distinct groups of raters; US college students and Amazon Mechanical Turk (AMT) workers. Specifically, the research used female facial representations of two racial origins (faces with black and white complexion) and either set of faces with artificial white noise or set of faces manipulated in sexual dimorphism. Authors report a racially-specific association between sexual dimorphism (facial femininity) and the perception of EDs. When noise was not applied to the images, both black and white female facial representations with feminised features were significantly more frequently perceived as suffering from Eating Disorders. However, when noise was applied on non-distorted (non-feminised) female faces, only black female faces that were perceived as ED-positive were at the same time identified as more feminine. The noise patterns that caused non-feminised (unmanipulated) white female faces to be perceived as ED-positive were not significantly associated with noise patterns that made their appearance more feminine. This result was based on two independent datasets of raters (College students and AMT workers). The authors also present an analysis of the effect of an image background and foreground darkness and root-mean-scale contrast on the perception of ED and femininity. The perception might have been affected by the difference in contrast and foreground/background luminance. The authors conclude that the perception of eating disorders is stereotypically associated with femininity only in black female faces. In white female faces, perceived femininity is not a cue to an ED.

The authors have brought convincing arguments that EDs are an issue in current US society. They also cited references to the studies showing that both students and clinicians are inaccurate in diagnosing the EDs in female faces. This accuracy may be further affected by stereotypes based on the sex typicality and ethnicity of patients. People stereotypically think that women of European origin possess eating disorders more frequently. The authors utilised two independent sets of raters (college students and AMT workers). At above-chance levels, the AMT workers and college students agreed on the diagnosis of ED in the stimuli faces. The authors also showed an important difference between white and black faces concerning the association between ED and PERCEIVED femininity. The goal to determine how race category (White vs Black) and perceived/manipulated femininity affect the likelihood of being perceived as a patient with ED-diagnosis has been fulfilled.

**Thank you.**

2. Primary objections

Nonetheless, author should address the following objections:

(a) In my opinion, authors should explain the evolutionary relevance and adaptive consequences of the fast and accurate perception of cues of an eating disorder in a female face. Any perceiver should struggle to maximise his or her fitness. Accordingly, it is adaptive to assess women’s impaired health and fertility accurately. Eating disorders have been shown to multiply the risk of death and impair fertility [1,2]. Therefore, I would appreciate a review section on the effects of eating disorders on perceived health and attractiveness. Perceived health seems to predict actual health partially [3,4]. Perceived attractiveness and healthiness are also essential cues in mate choice, mate success, and status negotiation [5,6]. People should perceive markers of eating disorders as cues of impaired health and fertility, low social status and unwanted social and mating partner.

Moreover, the noise the authors applied on the faces can be interpreted as an alteration of the texture of facial skin, which seems crucial for the perception of female youthfulness and fertility [7,8]. I think a paragraph on evolutionary and mate-choice consequences of impaired health would highlight the crucial importance of improving the knowledge of perception of eating disorders. It would also "anchor" the research topic in the paradigm of evolutionary psychology.

**We appreciate the reviewer’s points of trying to link our paper to evolutionary psychology. However, our paper is not meant to inform how individuals can use faces to assess for eating disorders diagnosis as a proxy for fitness for potential mates and how this varies by race. Our paper is meant to address how individuals can have perceptual judgements in line with implicit or explicit racial stereotypes in regards to who may have an eating disorder or not. This is important as these erroneous judgements may lead to people being more likely to overlook who may have an eating disorder and may need help. Our study is a first step for future research examining these potential visual biases in clinicians, which can lead to real world harm and disparities in treatment (e.g., a clinician overlooking an ED diagnosis in a Black individual due to their face not meeting their perceptual stereotypes for who has an ED). In addition, our society actually endorses body types associated with disordered eating behaviors such as being thin and lean at all costs (Thompson et al., 1999; Tylka, 2011). So, although evolutionarily human preferences select against eating disorders, our current societal expectations and preferences encourage the opposite. Thus, beyond this not being the intention of our paper, we believe that this argument does not fit the current state of the eating disorder literature.**

**Thompson, J. K., Heinberg, L. J., Altabe, M., & Tantleff-Dunn, S. (1999). Exacting beauty: Theory, assessment, and treatment of body image disturbance. Washington, DC: American Psychological Association.** [**https://doi.org/10.1037/10312-000**](https://doi.org/10.1037/10312-000)

**Tylka, T. L. (2011). Refinement of the tripartite influence model for men: Dual body image pathways to body change behaviors. *Body Image, 8*, 199-207.** [**https://doi.org/10.1016/j.bodyim.2011.04.008**](https://doi.org/10.1016/j.bodyim.2011.04.008)

(b) I do not understand why the authors use the method of adding a "noise" into artificial FaceGen faces to study “how does femininity co-vary with physiognomic cues related to the perception of disordered eating, and does this relationship vary by race?”. I would prefer to use natural faces of ED-positive and ED-negative individuals, get them rated on perceived femininity and presence of eating disorders. Then, I would analyse skin texture and facial shape to identify which cues in facial surface and shape make their appearance more ED-like. Following this, I would manipulate the faces to make them more ED-like and get those manipulated faces rated again. By the proposed attitude, I could identify cross-ethnical differences in various cues’ effect. If I ever wondered, which cues make people perceive someone ED-positive, I would use the methodology described above (texture and shape analysis, natural stimuli). And even if I used the method of "random noise", I would also try to identify, which patterns of noise drive the perception of ED-positivity and femininity! The face texture is of key importance for the perception of youthfulness, attractiveness and healthiness (see above). Nonetheless, authors do not identify which features of the texture drive the perception of ED and femininity. The most prominent "take-home message" is that "some pattern of noise is perceived as more feminine. Unlike in White women, in Black women, this noise is also perceived as associated with eating disorders". Still, the manuscript presents a good starting point concerning studying the perception of facial cues affecting the perception of Eating disorders. With regard to the PLOS ONE policy ("PLOS ONE uses peer review to determine whether a paper is technically rigorous and meets the scientific and ethical standards for inclusion in the published scientific record") the manuscript is in good order. However, authors apply a rare method, withouth further explaination (and justification). Authors might have been completely aware of the benefits of their method to a degree that these benefits are not mentioned (e.g. that it is easier than to get facial photos of people possessing ED-diagnosis). Nonetheless, such method might have been a result of a decision like "lets' use the RC method on the topic of EDs: the method of RC is novel and rare, the topic of ED seems important".

**We appreciate the reviewer’s suggestion regarding our choice of methodology. We have several responses to these points:**

**First, the reviewer suggests an alternative approach to identifying visual cues to the likelihood that someone has an eating disorder. Specifically, the reviewer suggests using real photographs of ED-positive and ED-negative individuals, and using these to identify facial features that are predictive of membership in these categories. We agree that this could be an interesting direction to pursue, but there are several reasons why we didn’t adopt this approach to address our research questions. There is, to our knowledge, not a sufficiently large existing database of white and black faces with accompanying eating disorder metadata (which the reviewer acknowledges), so we would need to first create such a resource to carry out the study that the reviewer suggests. Further, this approach addresses a different question than we set out to examine: While we were interested in understanding observers’ perception of eating disorder likelihood, what the reviewer is suggesting would instead be an examination of how physiognomy actually does correlate with eating disorder likelihood (if at all). Recently, physiognomic claims of this nature have been discussed with regard to the way in which they perpetuate stereotypes and reinforce prejudicial behavior against individuals based on their appearance. Broadly speaking, we agree that research promoting physiognomy is ethically questionable. In the current study, our goal was therefore not to try and establish a link between physiognomy and disordered behavior, but rather to measure what perceptual biases observers may maintain with regard to such a correlation. The latter helps elucidate how observers attempt to make predictions about complex behavior from facial appearance (even if these are not valid), while the former could be interpreted as an attempt to validate the perspective that one can “diagnose” ED by looking at someone.**

**Besides these issues, the approach that the reviewer suggests also requires that we identify image descriptors for surface texture and face shape that we can then compare across ED+ and ED- faces. These are not straightforward problems to solve. For example, while we agree with the reviewer that skin texture is an important cue to attractiveness, youthfulness, and other qualities that may covary with perceived ED likelihood, capturing these aspects of texture appearance quantitatively is not a solved problem by any means. The advantage of the reverse-correlation method is thus two-fold. (1) Rather than requiring a large database of existing images with accompanying metadata, we can instead use random noise to probe the perception of a large number of images based on a single parent image. (2) Instead of trying to develop useful descriptors for face shape and texture, the method yields a classification image per participant and condition, and the perception of that image can be examined by showing it to other observers.**

**Finally, far from being a rare technique, the reverse correlation method has been used widely in a number of sensory domains and applied to face recognition in a number of studies. We think it is therefore a useful tool that helps us address the research questions we sought to answer. We greatly appreciate the reviewer’s thoughtful comments regarding how to motivate our use of this technique, and we have included an expanded discussion of what the method offers that is distinct from some of the alternatives that the reviewer proposed in their commentary.**

(c) The section “Image statistics analysis” (p. 11-14), is, in my opinion, excessive. The authors attempted to determine the effect of differences of background and foreground darkness and also the effect of root-mean-square contrast of both background and foreground on the perceived attributes. The section seems to me to be based upon request or advice of other (previous) reviewers. Background and foreground may differ in contrast, darkness or both. One reason for face to be perceived feminine (and ED-positive) is to possess a texture (a pattern of foreground noise) that makes features more female-like (while keeping darkness and root-mean-scale of contrast constant in background and foreground). The other reason that some face would be perceived more feminine (and ED-positive) is that the noise in the foreground area was lighter than the noise in the background (or there were differences in RMS contrast between foreground and background). Therefore, I find it unnecessary to run such analyses. Authors should make the section shorter or transfer it into supplementary materials. It seems too long compared with the rest of the paper. It only tells: ‘The noise of the background/foreground of more feminine/ED-positive stimuli may be per se different from the noise in the background/foreground of less feminine/ED-negative stimuli'. It is, in my opinion, an inevitable property of the set of “noises” (and human perception). Authors may e.g. sum the results of these analyses into a table. If they decide to keep the section here, they should add (i) the definition of the “darkness” authors apply (e.g. a reverse scale to the CIELab Lightness?); (ii) the description of the variables (mean ± SD, range). Also, the name of the used statistical programme and a brief description of the analysis should be included in the manuscript (and the analysis code should be provided on-line). Last but not least, interested readers could run much more analyses, and I predict their courage would enable them to run all the analyses they wish (as soon as they will obtain the data), there is no need to tell them so explicitly.

**We agree that the full discussion is too lengthy, though to some extent these results do speak to the reviewer’s earlier suggestion to identify image statistics that correlate with perceived ED likelihood. The reviewer is right that there certainly must be some measurable difference between ED+ and ED- images, but that these specific measurements are different is not an inevitable consequence. As such, we have shortened the description of these results to focus more closely on the foreground/background intensity, which we think is a simple demonstration of how an image property that is known to correlate with perceived femininity varies in the classification images produced by our participants.**

3. Secondary objections

The minor issues are as follows (in order of appearance in the text):

Page 2 (abstract, last four lines): Feminisation is of importance for ED diagnosis; however, when the noise is applied, perceived femininity is unimportant for ED diagnosis in white faces.

**Thank you. We have edited the abstract to make this clear.**

On page 3 of the manuscript, authors state that “Subclinical eating disorders symptoms that do not fully cross the clinical threshold for a disorder can also be problematic”. They should cite a relevant source for this claim. I miss a definition of subclinical eating disorders symptoms. As an evolutionary psychologist, I would expect them to exist and affect the perceived traits.

**We have included a citation to support this point.**

The college students should present future clinicians? Or, is there, due to difficulties with ED-diagnosing, a need for attentive students who recognise there is “something wrong” with a schoolmate?

**Our focus was on naive observers so that we could investigate perceptual biases that ordinary individuals may maintain with regard to disordered eating behavior and appearance.**

The introduction, as a whole, seems to propose a “benchmark” study, which utilises the method of reverse correlation in an ecologically relevant context. However, the authors do not discuss (and justify) the psychological context in enough detail (see above).

**Please see our response in the previous section.**

Experiment 1

What does (on page 7) so-called “face space” stands for? I am aware of face space based upon facial landmarks and geometric morphometric (see, e.g.[9,10]). However, I would highly appreciate if the authors could bring a more detailed description of characteristics of FaceGen face space (e.g. a reference to previous studies that used FaceGen database). Also, the term “average faces” may describe a face that was attained by blending (or morphing) together with a set of faces [11,12]. Does “averageness” in FaceGen follow a similar method to create “average face”? In my opinion, there is no objection against using FaceGen stimuli; however, I cannot decide, whether the FaceGen faces “reflect systematic real-world category differences in face appearance” and whether “these capture sufficiently meaningful and representative appearance differences”. Are those claims based on some geometric morphometrics or other shape analyses? Otherwise, the authors should mention that using FaceGen faces may present a limitation (with regard to actual face diversity in the current US population).

**We have included additional discussion to describe what the term “face space” means in the context of the FaceGen model. Briefly, the model is based on laser-scanned data obtained from real faces, including 3D shape data and 2D albedo measurements. The resulting data has been used to create a dimensional model of 3D/2D appearance that reflects the variability that was present in the sample of scanned faces. Within that dimensional model, an average face can be defined as the face centered in the distribution of faces belonging to any target category (a white male average face, e.g.) The reviewer is right that the representativeness of that model depends critically on the diversity of that initial sample of faces, and we have included a reference to the white paper describing the characteristics of that sample. What we’re really trying to communicate here is that while FaceGen has important limitations, it is also based on data obtained from real people and thus does not reflect wholly artificial estimates of properties like femininity and averageness.**

Page 9-11: There is a full description of the analysis, and I think that authors use all the tests properly. Nonetheless, I miss the name of statistical software you use (e.g. “We did the test W using the function xyz in package zyx for R software (CITATION-OF-THE-PACKAGE)”).

**We apologize for this omission and have included this in the revised text.**

Page 10 (and 15): To my knowledge, it is possible to obtain more detailed information about AMT workers who completed the tasks. Authors should describe their country of origin, sex and average age.

**We apologize, but in our sample we did not collect this data and do not see a way to recover it from our data files.**

The data has been UNAVAILABLE from the link<https://osf.io/kbmz7/> (even though I possess an OSF account, and I requested the permission). Therefore, I could not observe the data, calculate the missing variable descriptions (e.g. mean +/- SD and range of darkness and RMS contrast), neither run the binomial, chi-quadrat tests and ANOVAs on my own. This needs to be fixed before the article release.

**We apologize for this oversight. We have confirmed that the OSF project is publicly available at present, so the data files and stimuli we describe in the text should be accessible now. If the reviewer encounters further problems trying to access these materials, we are happy to follow up as needed.**

Experiment 2 (page 15-17)

Authors should define the term “feminised” face in more detail. It may point either to (a) a result of artificial manipulation of faces based on scale connecting male and female facial configurations [9]; (b) a concept of perceived sex typicality, that may also be affected by non-shape cues [13]. Based upon information available, I predict that the authors followed the criteria and scale of femininity defined implicitly within the FaceGen database. However, I miss a definition of this scale: As mentioned previously, a “face space” is a rather diverse conceptual framework, which can be based upon 2D or 3D facial shape, skin contrast, colouration, and the allometric component of sexual dimorphism (and combination of those, like in FaceGen, most obviously). It is important to define the process of “feminisation” in more detail to allow other researchers to reply and extend the research based on other face databases. The statement that “varying faces according to FaceGen’s sense of femininity does correspond to meaningful appearance variation across male and female faces both in terms of 3D shape and 2D surface properties” is not sufficient. Authors should provide either (i) reference to the analysis of the 3D and 2D FaceGen faces’ properties which proves this; (ii) report of thier own analysis to support this claim.

**Thank you for this suggestion - this is relevant to the point we discussed above regarding the nature of the face space provided by FaceGen. Our revised description of the parameters of FaceGen’s model includes a discussion of the points the reviewer raises here. In brief, the FaceGen model is based on laser scans of male and female faces of different races, and feminisation in the resulting model is achieved by moving towards the portion of the estimated space that contains more female faces. This procedure includes image-level changes in skin tone and contrast, changes to the external contour of the face, and other changes to the shape of facial features like the eyes, nose, and mouth. Critically, all of these changes follow from the dimensional model established by the original sample of real male and female faces.**

Sometimes, authors do not follow formal rules for reporting the p-values: In the section “Image statistics analysis” (page 12), p = 0.035 is in neither case a “trend”. According to p = 0.035, you are allowed to “reject H0”.

**In this instance, the fact that we carried out multiple comparisons requires us to use an adjusted critical value of p=0.025. We have attempted to make this more clear in the revised text.**

In section “Experiment 2” (page 16-17) I miss a (two-tailed binomial) test to compare the proportions of trials in which feminised faces were chosen as looking more likely to have an ED diagnosis.

**We have included this in the revised text.**

In the Discussion (page 17-18) authors should discuss in more detail the fact that artificially feminised faces of both White and Black race were perceived more likely as having an ED diagnosis. When noise applied, only in black faces was there a significant association between perceived femininity and ED-diagnosis. Perceived femininity (based upon noise) and manipulated femininity tell a different story (the latter being not affected by the race of the face).

**Thank you for this comment. We have expanded our discussion of this point in the revised text.**

The language and wordings are easily-accessible.

**Thank you.**

Finally, I would like to thank you for that the authors brought me an option to read the current version of the report on the study. I hope that the authors have been able to understand all my remarks and questions and that they are able to cope with them to present their research report to the scientific community.

**Thank you for the thoughtful comments regarding our manuscript. We hope that we have addressed the points that you raised adequately and we will look forward to hearing from you regarding this draft.**
