# Supplementary material for: Facial femininity and perceptions of eating disorders: A reverse-correlation study
Source: PLoS One. 2021 Aug 6;16(8):e0255766. doi: 10.1371/journal.pone.0255766 (PMC8345843; doi:10.1371/journal.pone.0255766)
Supplement: Attachment — Submitted filename: Response to reviews.docx [file pone.0255766.s003.docx]

**Response to reviews:**

**We would like to thank the editor and the reviewers for their further comments on our revised manuscript. We are pleased to see that two of our reviewers indicated that the manuscript was acceptable for publication in its current form and we have incorporated the additional comments made by all of the reviewers into this current draft. Below, we respond to each of these comments individually to highlight how we have edited the text to address these remaining issues. The original review comments are in regular text and our responses are in bold throughout.**

Reviewer's Responses to Questions

Reviewer #1: The research report studied whether the diagnosis (perception) of eating disorder based solely on facial appearance is associated with facial femininity and whether the association is affected by race of the stimuli (Black or White). It used the reverse correlation technique.

In the previous (first) round of revisions, I already appreciated that the authors
(i) had improved and clarified any potentially misleading sections (e.g., explained that they did not study whether ED-diagnosing is anyhow accurate, described that they did not use a perspective of evolutionary psychology, and made clear that for serious reasons, they cannot use real ED-diagnosed individuals);
(ii) had extended section on the description of the reverse correlation technique;
(iii) had extended description on the FaceGen database, too.

After the second round of revisions, I have to confirm that none of those improvements was anyhow affected (worsened) by new modifications. Moreover, I appreciate that the section on the reverse correlation technique, which was good already, has been improved. I will remember that the paper contains such a section, and I would eventually refer to that when asked about the RC technique.

Furthermore, the authors added all missing references into the list and took my suggestions concerning several sentences, which were, in my opinion, misleading or hard to follow. The authors also uploaded potentially missing data to the online supplementary material. It allows following a "flow" of statistical analyses. Nonetheless, I have to declare that I am not an advanced user of the programs and tools that the authors used - JASP, Matlab, and MS Office Access. I am more familiar with R software.

Given that the paper focuses on two topics of increasing importance (eating disorders and racial stereotyping), concisely but sufficiently describes methods, reports and discuss the results correctly, and has been revised with regard to previously identified misleading or incorrect sections, I recommend it to final acceptance with no further revisions required. Apparent discrepancies in used font and poorly placed paragraphs within references would be, in my opinion, resolved by the final typeset in the journal.

**Thank you very much for these comments! We are very glad to see that our revisions addressed the comments this reviewer made in previous rounds of review.**

Reviewer #4: Basically, the current research seems to be academically meaningful. However, it seems that there are parts that need correction and completion of the research.

First, what is the operational definition of perceptual bias in this study? I wonder how the authors can prove that what they observe in this study corresponds to perceptual bias.

**Our operational definition of perceptual bias in the current study is observers’ tendency to link specific aspects of facial appearance to category labels: In this study, we are specifically interested in the link between perceived femininity (one aspect of facial appearance that is determined in part by pigmentation and contrast relationships in the face) and the categorization of faces according to the likelihood of an eating disorder diagnosis. As to whether or not we can truly “prove” that our results are the result of a perceptual bias, we would argue that proving causal mechanisms for behavioral results is generally extremely difficult. What we offer here (and take more care in the revised text to clarify) is evidence that observers appear to have different expectations about the covariation between feminine appearance and eating disorder likelihood as a function of stimulus race. Whether the causal mechanisms leading to this outcome are uniquely perceptual or cognitive, our task reveals that these expectations affect performance in a perceptual task. In the revised text, we have attempted to be more careful about our conclusions in this regard which we hope adequately addresses the reviewer’s point.**

Second, it is hard to understand what it means in reality for ordinary people to look at other people's faces and guess that they have eating disorders. In terms of diagnosis, clinically diagnosing eating disorders has no significant relationship with facial features. In particular, there are many forms of eating disorders, such as anorexia and bulimia, and it seems necessary to carefully explain how this potentially affected the research results.

**As we have described in the revised introduction, observers readily estimate a number of personality variables from face images despite many of these judgments having low validity. Across a large literature, observers have been demonstrated to make reliable judgments about trustworthiness, dominance, competence, and other aspects of mental health from face images, which we think indicates that this is indeed an aspect of face recognition that is relevant to real-world behavior and leads to quantifiable outcomes in a range of tasks that can be used to make inferences about the basis for those judgments. The reviewer is correct that there are not known links between facial appearance and eating disorder symptomology (which we are careful to point out in the text, contrasting our study with what has been dubbed “modern physiognomy” by experts in the ethics of face recognition), but this does not mean observers do not have expectations about correlations between appearance and behavior. We agree with the reviewer that the range of eating disorders could potentially be interesting to consider, but we chose to ask observers to make a judgment about disordered eating considered broadly to avoid the need to explain differences between, say, anorexia nervosa and binge eating disorder that might influence participants’ choices in our task. In the revised text we have edited the text to speak to both of these issues and make clear that it is not evidence of a valid link between facial appearance and disordered eating that we are pursuing, but rather observers’ potential beliefs about the same.**

Third, when participants are asked which of the two is more likely to have eating disorders by looking at the experimental stimulation faces, it is difficult to understand what criteria they made. How can we check if the participants understood the meaning of the task properly? Even if independent observers agreed with the results of in-lab participants, this does not address the question of what criteria they used in the evaluation process. Basically, it is a different task for the ordinary people to perform the task of choosing the more feminine of the two facial stimuli and the task of choosing the one with the potential for eating disorders.

**We respectfully disagree with the reviewer on this point. The reverse correlation technique specifically allows us to examine what aspects of facial appearance the observers were using to make this decision: The individual classification images show us what image templates correlate with the ED+ and ED- judgments per observer, and the independent ratings made by our additional participants allow us to examine how high-level variables like femininity also correlate with these judgments. Further, we suggest that the agreement between our two samples of participants regarding the likelihood of an eating disorder diagnosis in ED+ and ED- classification images is indeed a useful demonstration that both participant groups understood the task and understood it in similar ways. We also see no evidence in our data (or from post-testing debriefing) that participants did not understand the task properly: Observers’ classification images were categorized by independent observers at above-chance levels, suggesting that the image features used by both groups to assess the likelihood of an eating disorder were similar, and we also observed differences in specific image statistics across ED+ and ED- categories. Observers thus made reliable image-based judgments that led to measurable effects across our target categories. This 2AFC judgment is thus not any more suspect than other 2AFC judgments about facial appearance, especially when one considers the other examples of social categorization task we now describe in the revised introduction. While we agree with the reviewer that estimating the likelihood of an ED diagnosis is “a different task” than categorizing gender, it is also a different task that still leads to consistent behavior within and between individuals and did not lead our participants to express confusion with task requirements.**

Fourth, it seems difficult to interpret this study as providing evidence that “facial appearance may affect decisionmaking related to the estimated likelihood of health-related behaviors in others, in this case, disordered eating.” If another group of researchers constructed a research design to confirm the evidence that facial appearance can affect decisionmaking related to the estimated likelihood of eating disorders, I wonder if they would construct the same design as this study. The findings may not be a study of decision-making related to the estimated likelihood of eating disorders but simply reflecting stereotypes shared by ordinary people.

**Again, we respectfully disagree with the reviewer on this point. First, while we do not know if a hypothetical group of other researchers would come up with the same study design as we have used here, we also do not think this is a meaningful way to judge whether our work provides useful data or not. Different approaches to examine the same issue are valuable and we think this technique offers interesting insights that other designs would not. Indeed, as we describe in the introduction, reverse correlation tasks like this one have been used to establish similar links between facial appearance and various categorization judgments in many different cases. Our study is thus an application of an existing technique to a new problem domain, which we think does indeed address whether observers have expectations about how facial appearance may be related to health behaviors. We apologize, but we also do not understand distinction the reviewer makes between stereotypes shared by ordinary people (specifically with regard to what people who exhibit certain behaviors look like) and a link between facial appearance and decision-making. By the latter, we are simply referring to the outcomes we observed in our study: Observers made reliable decisions about behavior (eating disorders) based on what faces look like, which we think could reasonably be called a stereotype. We have assumed in preparing the revised text that the reviewer may have objected to our particular choice of words, and so we have edited the text to link our original wording to the language of stereotypes suggested by the reviewer. We hope this adequately addresses this point.**

In conclusion, although this is an interesting study, it seems necessary to refine the purpose and meaning of the study by considering the limitations of the study.

Reviewer #5: Referee report “Facial Femininity and Perceptions of Eating Disorders: A Reverse-Correlation Study” PONE-D-20-16005R2

Note: I use singular author throughout to refer to author/author(s)

Summary
This paper reports on two experiments (with three separate groups of participants involved) that elicited people to make choices regarding subjective evaluations of the appearance of faces, following a prompt. One experiment was a reverse correlation task (choosing between noise-adjusted face pairs according to a prompt), the other asked participants to characterize experimenter-manipulated face images (choosing between original and adjusted face according to a prompt). The faces and prompts varied on three dimensions: eating disorders (ED), gender, and race (white or black).

The author variously frames the main finding (or key hypothesis to be evaluated) as:
• “We hypothesized that observers would believe that faces that looked more feminine would also be more likely to have an ED diagnosis. Further, we hypothesized that this tendency might be affected by the race of the face under consideration due to additional observer biases affecting how femininity is perceived across racial categories.”
• “In particular, we examined whether or not perceived femininity was differentially linked to perceived likelihood of having an eating disorder diagnosis as a function of race.”
• “How does femininity co-vary with image features related to the perception of disordered eating, and does this relationship vary by race?”

The results are then described as:
• “We found that for Black faces, perceived femininity was associated with the perceived likelihood of having an eating disorder diagnosis. In our second experiment, directly manipulating femininity to influence eating disorder categorization did not lead to differences in the outcome for Black and White faces, though we did observe a trend favoring a larger impact of feminization on the rate at which feminized Black faces were categorized as more likely to have an eating disorder diagnosis.”
• “For Black faces, on the other hand, since their race violates the stereotype of who is more likely to have an eating disorder, assessors may tend to focus more on the relevant characteristic they have that goes with the stereotype - being female. As being female becomes the characteristic of focus for Black faces, an increase in facial femininity leads to a stronger association with eating disorder.”

The article is motivated by the observation that a distinct population, college students, on average neither recognizes ED symptoms, nor the need for ED treatment, nor is able to “consistently identify what constitutes disordered eating”. Moreover, college students (?) likely have racial stereotypes of EDs.

**Thank you for this summary – this indeed largely captures the design and results of our study.**

Overall Evaluation
The paper is well-written, the methods are appropriate, the analysis is competent. Authors offer a clear and concise presentation. The findings are somewhat mixed: at the end this reader was not sure whether to come away thinking, “race definitely matters in mediating people’s association with ED and femininity” or to come away thinking “probably race does not matter much in mediating people’s association with ED and femininity.”

A significant missing element in the protocol/data is an identifier of race for the experiment respondents: while Black v. white face might condition the correlation between femininity and ED, presumably the race-identification of the participants (as Black or white, or other ascribed or self-ascribed ethnic identities) would matter significantly. Would a population of Black persons likely have a correlation between femininity and ED similar to that of a population of white persons, for images of Black and white faces? This strikes me as unlikely, and yet the identities of the participants are not mentioned (only their gender is noted but not used as a covariate in the analysis). Perhaps there were no Black students in the college sample? The Amazon MechTurk tasks in Experiment 1 and Experiment 2 were done by respondents (unclear how many), and their identity status appears not to be a covariate in the analysis.

**We apologize for this omission – the sample of students who completed the classification image experiment was predominantly comprised of white students, which we include in the revised draft. We agree that including participant race as a covariate would be an excellent analysis, recruiting a sufficient sample of Black students is typically challenging at our institution given the demographics of the student population. With regard to our Mechanical Turk experiments, we chose not to collect race information from these participants. In the revised text we make this explicit, clarify the number of participants in each task, and discuss how observer race could affect these judgments.**

A related area of ambiguity (for this reader) was who was expected to be the population about which the research could be generalized. In first paragraph, this appears to be college students, but then the second paragraph mentions clinicians having stereotypes. Then bottom of p. 3 becomes more general, mentioning “people.” On p. 6 and 7 the relevant population is called “naïve observers.” Then in methods, the Experiment 1 RC task was done by 28 college students from North Dakota (no mention of how identity backgrounds might matter except to mention that clinicians were excluded). The two Amazon MechTurk samples were presumably not college students. So it is unclear who constitutes the population of interest. Amazon MechTurk are hardly a random sample of a well-defined population, and author should perhaps consult the expanding literature on how to generalize from Amazon MechTurk samples.

**We apologize for the confusion. First, our brief mention of clinicians was not intended to be a claim about the generality of our results, but rather a broader statement about why understanding the nature of these biases could be relevant to real-world contexts. Reviewer #4, for example, doubted whether or not estimating the likelihood of an eating disorder diagnosis from appearance was even a reasonable task to imagine, and we suggest that the clinical context is an important way to respond to this question. Second, Experiment 1 was indeed completed using a sample of college students, with no particular aspects of “identity background” constrained or measured. While we agree that the use of AMT in our subsequent tasks does represent a different (and likely more variable) population, this is increasingly common across a range of cognitive tasks and we think does not pose any major challenges to our main conclusions. We do agree that it is important to highlight that the judgments made in Experiment 1 were made by a specific participant group (college students in the US) and this may not be representative of the performance we might see in a different or broader group. The revised text includes a discussion of this point. We also discuss what conclusions we can draw from our AMT sample with regard to generality, trying to emphasize a conservative interpretation of our results.**

Presentation of results (some questions and suggestions for improvement)
There are essentially three results in the paper.
1. “The [28 pairs of] classification images created by in-lab participants were thus reliably interpreted by independent observers as looking more likely to have an eating disorder across both White and Black faces, but race affected how perceived femininity covaried with the appearance characteristics associated with disordered eating by our first group of observers.”
a. Unclear here whether 560 participants each scored one pair? Or whether 40 Amazon MechTurk participants each scored the 28 pairs? The paper says, “We collected a total of 560 independent judgments for each question for both.” This makes it sound like 560 different participants? Is there no information about these participants?

**Yes, we asked for 560 independent judgments and did not collect demographic data. While this means we cannot include covariates in our analysis, our results simply collapse across whatever variability is in the data to describe the main results as simply as possible.**

b. White and Black classification images +ED were correlated with the evaluation of separate raters that +ED were somewhat more likely to have an eating disorder. Black +ED faces were more likely to be perceived as more feminine than –ED pair.
2. The differences in reverse correlation +ED were not due to differences in background or face luminance (and nice validation exercise).
a. There appears to be a mistake in the text: on p. 15 “(t (27) = -2.21, p = 0.035)” so normally this indicates rejecting the null hypothesis, but the text states “nor Black face had systematically darker backgrounds for ED+ face images compared to ED- face images”

**We would like to be cautious in terms of our interpretation of this difference given the multiple comparisons we included here. Applying a Bonferroni correction our data requires an alpha of 0.025 rather than 0.05, which means this result does not reach significance. We have clarified this in the revised text.**

b. One might wonder though whether “mottling” of face is/was relevant for ED choice?

**This is an interesting issue, but difficult for us to address meaningfully using the current data.**

3. 400 Amazon Mechanical Turk workers each examined one image pair and rated it. The raters preponderately found that the femininized faces were more likely to have an eating order diagnosis. Black and white faces were only marginally different from each other.

Smaller questions
Fig. 2 – The average classification images – is this average of the averages- just to be clear? Or is it one of the 28 individual CI?

**Yes, this is an average of averages.**

How many of Amazon MechTurk who did task may have been repeat workers? That is, could they not have multiple accounts and so the same person did many responses?

**We do not know a clear way to determine whether or not someone created multiple accounts to carry out different judgments. We suggest this is another reason to be skeptical of our ability to collect meaningful demographic data about our AMT workers and is part of the reason we chose to collect and analyze this data in the aggregate.**

Comment
More modesty about what RC does? “the experimenter can obtain an image that reflects what properties of the noise were correlated with perceived membership in that category.” Could be “the experimenter can obtain an image that might possibly reflect some patterns of the noise correlated with perceived membership in that category.” (the possibly is because some of the correlations might be spurious as there are very large number of patterns (i.e., quite multidimensional) and simple patterns might be correlated when the “true” pattern that generates correlation is more complex.

**We have added the word “possibly” to address this important point.**

**Once again we would like to thank our reviewers for their continued commentary on this 3^rd^ revision of our manuscript. We hope that our revisions here have addressed their concerns and appreciate their constructive comments. We feel that the manuscript has been further strengthened by their thoughtful questions and observations.**
